# Budlein A, a Sesquiterpene Lactone From *Viguiera robusta*, Alleviates Pain and Inflammation in a Model of Acute Gout Arthritis in Mice

**DOI:** 10.3389/fphar.2018.01076

**Published:** 2018-09-25

**Authors:** Victor Fattori, Ana C. Zarpelon, Larissa Staurengo-Ferrari, Sergio M. Borghi, Tiago H. Zaninelli, Fernando B. Da Costa, Jose C. Alves-Filho, Thiago M. Cunha, Fernando Q. Cunha, Rubia Casagrande, Nilton S. Arakawa, Waldiceu A. Verri

**Affiliations:** ^1^Laboratory of Pain, Inflammation, Neuropathy, and Cancer, Department of Pathology, Londrina State University, Londrina, Brazil; ^2^AsterBioChem Research Team, School of Pharmaceutical Sciences of Ribeirão Preto, University of São Paulo, Ribeirão Preto, Brazil; ^3^Laboratory of Inflammation and Pain, Department of Pharmacology, Ribeirão Preto Medical School, University of São Paulo, Ribeirão Preto, Brazil; ^4^Department of Pharmaceutical Sciences, Center of Health Sciences, Londrina State University, Londrina, Brazil

**Keywords:** knee pain, joint pain, gout treatment, rheumatic disease, experimental arthritis, NLRP3 inflammasome, analgesia, sesquiterpene lactones

## Abstract

**Background:** Gout is the most common inflammatory arthritis worldwide. It is a painful inflammatory disease induced by the deposition of monosodium urate (MSU) crystals in the joints and peri-articular tissues. Sesquiterpene lactones (SLs) are secondary metabolite biosynthesized mainly by species from the family Asteraceae. It has been demonstrated that SLs present anti-inflammatory, analgesic, antitumoral, antiparasitic, and antimicrobial activities. In this study, we aimed at evaluating the efficacy of the SL budlein A in a model of acute gout arthritis in mice.

**Methods:** Experiments were conducted in male Swiss or male LysM-eGFP mice. Animals were treated with budlein A (1 or 10 mg/kg) or vehicle 30 min before stimulus with MSU (100 μg/10 μL, intra-articular). Knee joint withdrawal threshold and edema were evaluated using electronic von Frey and caliper, respectively, 1–15 h after MSU injection. Leukocyte recruitment was determined by counting cells (Neubauer chamber), H&E staining, and using LysM-eGFP mice by confocal microscopy. Inflammasome components, *Il-1β*, and *Tnf-α* mRNA expression were determined by RT-qPCR. IL-1β and TNF-α production (*in vitro*) and NF-κB activation (*in vitro* and *in vivo*) were evaluated by ELISA. *In vitro* analysis using LPS-primed bone marrow-derived macrophages (BMDMs) was performed 5 h after stimulation with MSU crystals. For these experiments, BMDMs were either treated or pre-treated with budlein A at concentrations of 1, 3, or 10 μg/mL.

**Results:** We demonstrated that budlein A reduced mechanical hypersensitivity and knee joint edema. Moreover, it reduced neutrophil recruitment, phagocytosis of MSU crystals by neutrophils, and *Il-1β* and *Tnf-α* mRNA expression in the knee joint. *In vitro*, budlein A decreased TNF-α production, which might be related to the inhibition of NF-κB activation. Furthermore, budlein A also reduced the IL-1β maturation, possibly by targeting inflammasome assembly in macrophages.

**Conclusion:** Budlein A reduced pain and inflammation in a model of acute gout arthritis in mice. Therefore, it is likely that molecules with the ability of targeting NF-κB activation and inflammasome assembly, such as budlein A, are interesting approaches to treat gout flares.

## Introduction

Gout is the most common inflammatory arthritis worldwide. It is a painful inflammatory disease induced by the deposition of monosodium urate (MSU) crystals in the joints and peri-articular tissues (Rees et al., 2014; Dalbeth et al., 2016; Fattori et al., 2016). These crystals are recognized by resident cells, such as macrophages, which produce IL-1β in an NLRP3 inflammasome-dependent manner (Martinon et al., 2006). The production of IL-1β in the knee joint is the initial step for the recruitment of neutrophils that further produce hyperalgesic mediators, such as reactive oxygen species (ROS), TNF-α, IL-1β, and PGE_2_ (Popa-Nita et al., 2007; Amaral et al., 2012; Fattori et al., 2016). In general, gout arthritis is well controlled if properly managed, but, despite that, gout can trigger acute flares that are usually the reason patients seek medical attention. In fact, acute gout flares are recognized as one of the most painful experiences, on the same level as childbirth or visceral colic (Rees et al., 2014). Gout flares in humans are self-limited with spontaneous resolution within 7–10 days (Martin and Harper, 2010), whereas in mice, the resolution occurs within 24–36 h, given MSU crystals are rapidly destroyed by uricases (Amaral et al., 2012; Galvao et al., 2017). However, if left untreated, continuing MSU crystals deposition not only causes further flares but also, and importantly, promotes irreversible joint damage with chronic symptoms and disability (Rees et al., 2014). In fact, patients with advanced gout often present chronic joint pain, swelling, movement limitation, and recurrent flares (Dalbeth et al., 2016). Currently, the management of gout flares lies in the use of non-steroidal anti-inflammatory drugs (NSAIDs), colchicine, corticoids, or biological agents (Rees et al., 2014; Dalbeth et al., 2016). Nonetheless, these drugs often cause severe side effects and lack of safety in patients with comorbidities (Rees et al., 2014; Dalbeth et al., 2016). Thus, novel drugs with fewer side effects are still needed.

Sesquiterpene lactones (SLs) are secondary metabolites biosynthesized mainly by species from the plant family Asteraceae (Hohmann et al., 2016; Padilla-Gonzalez et al., 2016). SLs are present in, for example, lettuce and chicory (*Lactuca sativa* and *Chicorium intybus L*.) and represent an important part of human diet (Chadwick et al., 2013). It has been demonstrated that SLs present anti-inflammatory, analgesic, antitumoral, antiparasitic, and antimicrobial activities (Hohmann et al., 2016). Concerning gout, treatment with ethanolic extract of *Lychnophora passerina* (Mart. ex DC.) Gardner (Brazilian *Arnica*, Asteraceae) containing the SL goyazensolide (the major metabolite) ameliorated oxonate-induced hyperuricemia in mice, indicating that they could be also used as urate-lowering therapies (de Albuquerque Ugoline et al., 2017). Budlein A is an SL with analgesic (Valerio et al., 2007; Zarpelon et al., 2017) and anti-inflammatory properties (Valerio et al., 2007; Arakawa et al., 2008; Nicolete et al., 2009; Knob et al., 2016; Zarpelon et al., 2017) related to the inhibition of pro-inflammatory cytokines and neutrophil recruitment. Mechanistically, SLs can inhibit NF-κB activation (Lyss et al., 1998; Siedle et al., 2004; Zhao et al., 2015; Widen et al., 2017; Zarpelon et al., 2017) or even direct target inflammasome assembly (Juliana et al., 2010; Mathema et al., 2012). Given the pharmacological properties of budlein A, in this study we investigated the efficacy of budlein A extracted from *Viguiera robusta* (also known as *Aldama robusta*) (Gardner) E.E. Schill & Panero (Asteraceae) in a model of MSU-induced gout arthritis in mice.

## Materials and Methods

### Animals

The experiments were performed on healthy male Swiss, C57BL/6, or LysM-eGFP C57BL/6 background mice weighing between 20 and 25 g and with 8 weeks of age. Mice were housed in standard clear plastic cages in temperature-controlled room (22–25°C), with access to water and food *ad libitum*. All experiments were conducted in accordance with animal care and handling procedures of the International Association for Study of Pain (IASP) and with the approval Londrina State University Ethics Committee on Animal Research and Welfare (protocol number 14544.2013.46). All efforts were made to minimize the number of animals used and their suffering. Animal studies are reported in accordance with the ARRIVE guidelines for reporting experiments involving animals (Kilkenny et al., 2010; McGrath and Lilley, 2015). Euthanasia was performed by isoflurane anesthesia (5% in oxygen using a precision vaporizer) followed by decapitation as a confirmation method. A total of 280 mice were used in this study. No animals were excluded from statistical analysis.

### Plant Material

**FIGURE 1:**
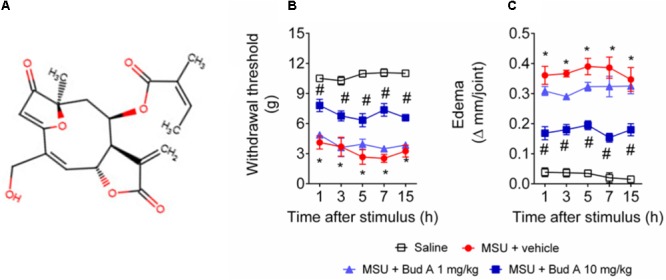
Budlein A decreases MSU-induced mechanical hypersensitivity and knee joint edema. Chemical structure of Budlein A is represented in panel **(A)**. The molecule was drawn using Marvin JS, MarvinSketch in JavaScript. Mechanical hypersensitivity **(B)** and knee joint edema **(C)** were evaluated 1, 3, 5, 7, and 15 h after the injection of MSU crystals using electronic von Frey and caliper, respectively. ^∗^*p* < 0.05 vs. saline group; ^#^*p* < 0.05 vs. 0 mg/kg group, two-way repeated measures ANOVA followed by Tukey’s *post hoc*.

Budlein A (**Figure 1A**) was isolated and purified from *V. robusta* (also known as *A. robusta*), which was collected in April 2001 by N. S. Arakawa (Batatais, S 20°97′16″, W47°49′90″, SP). The plant material was identified by J. N. Nakashima at the Biology Department, Federal University of Uberlândia, Uberlândia, MG, Brazil. A voucher specimen (FBC#105) was deposited at the herbarium of the Department of Biology, University of São Paulo, Ribeirão Preto, SP, Brazil under the code SPFR 07155. Two and a half kilograms of the air-dried leaves of *A. robusta* were placed in an Erlenmeyer flask and extracted by sonication with dichloromethane (room temperature, 10 min). The residue was filtered with common filter paper and the solvent was removed under vacuum. After that process, 14 g of dried crude extract was obtained, which was analyzed by infrared (IR) spectroscopy. The presence of a strong band at 1.760 cm^-1^ in the spectrum corresponded to the carbonyl stretching of γ-lactones, indicating the presence of SLs in the extract (Hohmann et al., 2016). To remove pigments and fats, the extract was dissolved in methanol–water (4:1) and successive partition was made with *n*-hexane, dichloromethane, and methanol. After solvent evaporation (under vacuum) 3.1, 4.0, and 6.5 g of organic soluble residues were obtained. After IR spectral analysis, a strong band of γ-lactones was observed in the spectrum of the dichloromethane residue. Nine fractions were observed by thin-layer chromatography (TLC) after the residue was fractioned using vacuum liquid chromatography (silica gel, Merck, and *n*-hexane:ethyl acetate, increasing polarity). γ-Lactones were detected in fractions 6 (1,042 mg) and 7 (725 mg) as observed *via* IR spectral analysis. Pure budlein A (500 mg, white crystals) was obtained from fraction 6 after exhaustively washing with cold ethanol. Its chemical structure was determined using IR and nuclear magnetic resonance (NMR) spectrometry (^1^H and ^13^C), as well as comparison with authentic sample and data reported in the literature (Da Costa et al., 2001). The purity of budlein A was determined between 95 and 98% by chromatographic and spectrometric methods. TLC was carried out using several eluent systems and two spray reagents (1% vanillin-sulfuric acid or concentrated sulfuric acid). A high-performance liquid chromatography analysis was made using methanol–water 55:45 or acetonitrile–water 65:35 as mobile phase, a reversed phase (ODS) analytical column, flow rate 1.0 or 1.3 mL/min, and UV detection at λ_max_ 225 and 265 nm, as described elsewhere (Da Costa et al., 2001). Only one compound was detected in the chromatographic analyses. The ^13^C NMR spectrum of budlein A showed the characteristics peaks of carbon atoms that are assigned to its structure (**Figure 1A**) (Da Costa et al., 2001; Valerio et al., 2007).

### Preparation of the MSU Crystals

Monosodium urate crystals were prepared by dissolving uric acid (800 mg) in boiling ultrapure water (155 mL) containing 1N NaOH (5 mL), as described previously (Ruiz-Miyazawa et al., 2017). The pH of the solution was adjusted to 7.2 and it was cooled at room temperature. The crystals were collected by centrifugation (3,000 *g*, 2 min, 4°C), evaporated, and sterilized by heating at 180°C for 2 h. MSU crystals were stored in a sterile microtube until use.

### Induction of MSU-Induced Knee Joint Inflammation

Knee joint pain and inflammation were induced by the intra-articular (i.a.) administration of MSU (100 μg/10 μL) into the right knee joint of mice under isoflurane anesthesia. Saline animals received an i.a. injection of sterile saline also under isoflurane anesthesia (10 μL) (Ruiz-Miyazawa et al., 2017).

### Evaluation of Knee Joint Hypersensitivity

The mechanical hypersensitivity of the femur-tibial joint evaluated using an electronic von Frey apparatus (Insight instruments, Ribeirao Preto, SP, Brazil). In a quiet, temperature-controlled room, mice were placed in acrylic cages (12 cm × 10 cm ×17 cm) with wire grid floors, at least 45 min before the start of testing. Mice were habituated during four consecutive days in 60 minutes sessions prior to the behavioral testing. To exclude the subcutaneous effect for the evaluation of knee joint hypersensitivity, it used a large polypropylene tip (4.15 mm^2^ area size) (Guerrero et al., 2006). An increased perpendicular force was applied to the central area of the plantar surface of the hind paw to induce flexion of the femur-tibial joint. Positive response was considered after withdrawal of the hind paw. After withdrawal, the intensity of the pressure was automatically recorded, with the final response value being obtained by the average of three measurements. The intensity of the force applied (in grams) was recorded using a digital algesimeter. The mechanical force was applied when the animals were quiet and with the four paws on the grid floor. The upper limit pressure was 15 g. The test was performed for 1, 3, 5, 7, and 15 h after MSU. The investigators were blinded to the treatment.

### Knee Joint Edema Evaluation

Knee joint edema was determined using a caliper (Mitutoyo) before (zero time), and 1, 3, 5, 7, and 15 h after MSU. Knee joint edema was determined for each mouse by the difference between the time point indicated on figures and the baseline value (zero time). The edema value is represented as Δmm/joint. The investigators were blinded to the treatment.

### *In vivo* Leukocyte Migration

Knee joint wash was collected 15 h after MSU injection for determination of leukocyte recruitment (Ruiz-Miyazawa et al., 2017). Briefly, articular cavities were washed 3 times with 3.3 μL of saline with 1 mM EDTA, then, diluted to a final volume of 50 μL with PBS/EDTA to evaluate leukocyte migration. The total number of leukocytes was determined in a Neubauer chamber diluted in Turk’s solution 1:2 to lyse the erythrocytes. Differential cell counts were determined in Rosenfeld stained slices using a light microscope and results are expressed as the number of mononuclear cells or neutrophils per cavity.

### Histopathological Analysis

Knee joint was dissected 15 h after MSU injection, fixed with 10% paraformaldehyde in PBS, and then decalcified for 10 days with EDTA and embedded in paraffin for histological analysis. The paraffin sections were stained with hematoxylin and eosin for conventional morphological evaluation using a light microscope on 10× and 40× objectives. Results are expressed as leukocytes infiltrate (cell/field) counted at the inflammatory foci using the 40× objective. The dimension used for the analysis of the slices was 569 × 633 pixels (Ruiz-Miyazawa et al., 2017).

### Fluorescence Assay

Knee joint wash of LysM-GFP mice was collected in sterile slides 15 h after MSU injection. DAPI fluorescent stain was added to slides for localization of nucleus in each sample. The representative images and quantitative analysis were performed using a confocal microscope (SP8, Leica Microsystems, Mannheim, Germany). The intensity of fluorescence was quantified in randomly selected fields. Results are presented as the percentage of GFP fluorescent intensity. The number of LysM-eGFP cells in contact with MSU crystals was quantified in randomly selected fields by counting the colocalization between those cells and MSU crystals using the 63× objective. Results are presented as the number of phagocytosis of MSU crystals.

### Myeloperoxidase (MPO) Activity

Neutrophil recruitment to the knee joint was evaluated through the quantification of the enzyme MPO activity, as previously described (Fattori et al., 2017). Briefly, mice were terminally anesthetized, and the knee joint was dissected into 200 μL of 50 mM K_2_HPO_4_ buffer (pH 6.0) containing 0.5% HTAB and then homogenized in ice-cold Tissue-Tearor (Biospec). After that, homogenates were centrifuged (16,100 *g*, 2 min, 4°C), and the supernatants were collected. Supernatant aliquots of 30 μL were placed in a 96-well plate and mixed with 200 μL of 50 mM K_2_HPO_4_ buffer (pH 6.0), containing 0.0167% *ortho*-dianisidine dihydrochloride and 0.05% H_2_O_2_. The absorbance was determined after 15 min at 450 nm (Multiskan GO microplate spectrophotometer, ThermoScientific, Vantaa, Finland). The MPO activity of samples was compared to a standard curve of neutrophils and presented as MPO activity/mg of protein.

### Reverse Transcription and Quantitative Polymerase Chain Reaction (RT-qPCR)

Knee joint was dissected into TRIzol^®^ reagent 15 h after MSU injection. Total RNA was isolated according to manufacturer’s directions. The purity of total RNA was measured with a spectrophotometer and the wavelength absorption ratio (260/280 nm) was between 1.8 and 2.0 for all preparations. Reverse transcription of total RNA to cDNA and qPCR were carried out using GoTaq^®^ 2-Step RT-qPCR System (Promega) and specific primers (Applied Biosystems^®^). Primers sequences used were the following: *Tnf-α*: forward: 5′-TCTCATCAGTTCTATGGCCC-3′, reverse: 5′-GGGAGTAGACAAGGTACAAC-3′; *Pro-Il-1β* (*Il-1β*): forward: 5′-GAAATGCCACCTTTTGACAGTG-3′, reverse: 5′-TGGATGCTCTCATCAGGACAG-3′; and *Gapdh:* forward: 5′-CATACCAGGAAATGAGCTTG*-3*′, reverse: 5′-ATGACATCAAGAAGGTGGTG-3′. The relative gene expression was measured using the comparative 2-(^ΔΔCt^) method. The expression of *Gapdh* mRNA was used as a reference gene to normalize data.

### Preparation of Bone Marrow-Derived Macrophages (BMDMs) and Inflammasome Activation Assay

Femora and tibiae of C57BL/6 mice (8 weeks old) were aspirated with RPMI 1640 media. Bone marrow cells were cultured in RPMI 1640 medium containing 10% FBS and 15% L929 cell conditioned medium. BMDMs were harvested at day 7 and plated at the density of 1.5 × 10^5^ cells/well in 96-well plate. BMDMs were stimulated with 500 ng/mL of lipopolysaccharide (LPS) from *Escherichia coli* (Santa Cruz Biotechnology) and 3 h later treated with 450 μg/mL of MSU to stimulate NLRP3 inflammasome activation as described previously (Martinon et al., 2006; Ruiz-Miyazawa et al., 2017). BMDMs were treated with budlein A at 1, 3, or 10 μg/mL 30 min before MSU stimulation. Supernatants were collected 5 h after MSU stimulation and IL-1β concentration quantitated by ELISA. To evaluate TNF-α production, BMDMs were pre-treated with budlein A at 1, 3, or 10 μg/mL 30 min before 500 ng/mL of LPS. Supernatants were also collected 5 h after MSU stimulation. Lactate dehydrogenase release in the supernatant was used as a marker of cellular viability.

### Cytokine Production

For cytokine production, supernatant of BMDM culture was collected 5 h after MSU. TNF-α and IL-1β levels were determined by ELISA using eBioscience kits (eBioscience, San Diego, CA, United States) accordingly with manufacturer instructions. Reading was performed at 450 nm (Multiskan GO Microplate Spectrophotometer, Thermo Scientific, Vantaa, Finland). The results were expressed as picograms (pg) of each cytokine per mL.

## Nf-κB Activation

The determination of NF-κB activation in knee joint was performed accordingly with manufacturer instructions. For the *in vivo* assay, knee joint was dissected into ice-cold lysis buffer (Cell Signaling Technology, Beverly, MA, United States) 15 h after MSU injection. *In vitro*, cell lysates were collected into ice-cold lysis buffer (Cell Signaling Technology, Beverly, MA, United States) 5 h after MSU. The homogenates or cell lysates were centrifuged (16,100 *g* × 10 min × 4°C) and the supernatants used to assess the levels of total and phosphorylated NF-κB p65 subunit by ELISA using PathScan kits #7836 and #7834, respectively (Cell Signaling Technology, Beverly, MA, United States). The results are presented as the sample ratio (phospho-p65/total-p65) measured at 450 nm.

### Experimental Protocol

Male Swiss mice were treated with budlein A (1 or 10 mg/kg, diluted in 20% Tween 80 in saline, p.o.) 30 min before i.a. injection of MSU (100 μg/10 μL). Mechanical hypersensitivity (electronic von Frey) and knee joint edema (caliper) were evaluated 1, 3, 5, 7, and 15 h after MSU. Fifteen hours after MSU injection, knee joint wash was collected for determination of leukocyte recruitment. Based on these dose-response data, the dose of 10 mg/kg was chosen for the following experiments: neutrophil migration to the knee joint wash using LysM-eGFP mice (confocal microscopy), histopathological analysis (H&E staining), *Il-1β, Tnf-α, Nlrp3, Asc*, and *Caspase-1* mRNA expression by RT-qPCR, and NF-κB activation by ELISA. In all *in vivo* analysis, samples were dissected 15 h after MSU injection. For *in vitro* analysis, BMDMs were treated with budlein A at the concentrations of 1, 3, or 10 μg/mL 30 min before stimulus with MSU. Supernatants were collected 5 h after MSU and IL-1β concentration was determined by ELISA. To evaluate TNF-α production and NF-κB activation, BMDMs were pre-treated with budlein A at 1, 3, or 10 μg/mL 30 min before 500 ng/mL of LPS. Supernatants and cell lysates were also collected 5 h after stimulus with MSU. These time points and protocols were based on previous standardization of our laboratory (Ruiz-Miyazawa et al., 2017).

### Statistical Analyses

Results are presented as means ± SEM of measurements made of six mice in each group per experiment, except for those involving BMDMs. For those experiments, results are expressed as mean ± SEM, *n* = 6 wells. Experiments were conducted twice. Data were analyzed using the software GraphPad Prism 6.01. Two-way repeated measures analysis of variance (ANOVA), followed by Tukey’s *post hoc*, was used to compare all groups and doses when responses were measured at different time points after the stimulus injection. When an analysis was performed at a single time point, one-way ANOVA followed by Tukey’s *post hoc* was used. Statistical differences were considered significant when *P* < 0.05.

## Results

### Budlein A Reduces MSU-Induced Mechanical Hypersensitivity and Knee Joint Edema

First, it was addressed the best analgesic dose of budlein A (**Figure 1A**). The analgesic effect was addressed using budlein A at the doses of 1 or 10 mg/kg, as previously described (Zarpelon et al., 2017). We found that budlein A at the dose of 10 mg/kg reduced MSU-induced mechanical hypersensitivity as observed by an increase in the knee joint withdrawal threshold (**Figure 1B**) and reduction in the knee joint edema (**Figure 1C**).

### Budlein A Inhibits MSU-Induced Mononuclear Cell and Neutrophil Recruitment to the Knee Joint

**FIGURE 2:**
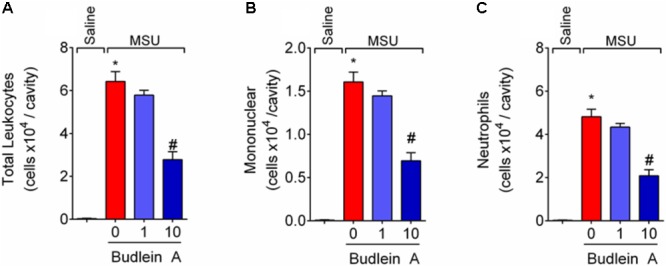
Budlein A inhibits MSU-induced mononuclear cell and neutrophil recruitment to the knee joint. Fifteen hours after the injection of MSU crystals, knee joint wash was collected for determination of total leukocytes **(A)**, neutrophils **(B)**, and mononuclear cells **(C)** using Neubauer chamber **(A)** and Rosenfeld stained slices **(B,C)**. ^∗^*p* < 0.05 vs. saline group; ^#^*p* < 0.05 vs. 0 mg/kg group, one-way ANOVA followed by Tukey’s *post hoc*.

**FIGURE 3:**
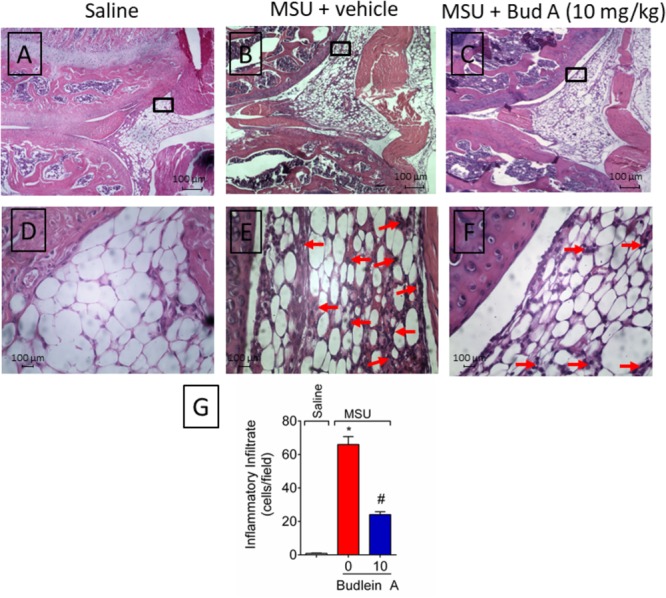
Budlein A reduces MSU-induced synovitis. Fifteen hours after the injection of MSU crystals, knee joint was dissected for histopathological analysis by H&E staining using a light microscope [Original magnification 10× **(A–C)** and 40× **(D–F)**]. The representative total score of inflammatory cells counted in the 40× objective is presented in panel **(G)**. ^∗^*p* < 0.05 vs. saline group; ^#^*p* < 0.05 vs. 0 mg/kg group, one-way ANOVA followed by *post hoc*.

**FIGURE 4:**
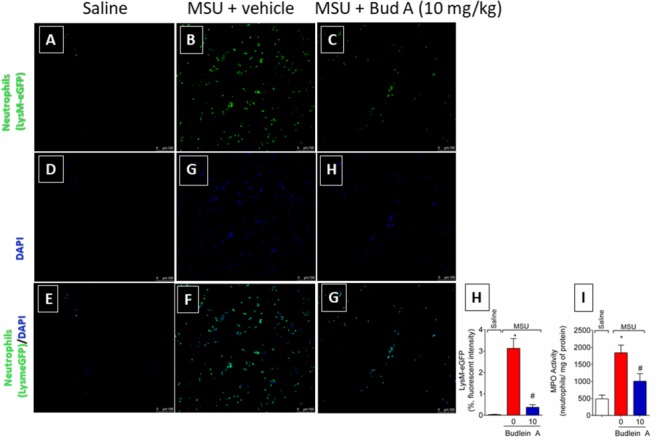
Fifteen hours after the injection of MSU crystals, knee joint wash of LysM-eGFP mice was collected for quantification of fluorescence intensity [Original magnification 20×, panels **(A-G)**] using a confocal microscope. Percentage of fluorescence is represented in panel **(H)**. Knee joint of Swiss mice was dissected for determination of neutrophil recruitment assessed by determination of MPO activity **(I)** by a colorimetric method. ^∗^*p* < 0.05 vs. saline group; ^#^*p* < 0.05 vs. 0 mg/kg group, one-way ANOVA followed by Tukey’s *post hoc*.

Recruited leukocytes, especially neutrophils, play important role in the genesis of pain in rheumatic diseases (Dalbeth et al., 2016; Fattori et al., 2016). Thus, it was evaluated the efficacy of budlein A in inhibiting MSU-induced leukocyte recruitment to the knee joint. Treatment with budlein A at 10 mg/kg reduced MSU-induced total leukocyte (**Figure 2A**), mononuclear cell (**Figure 2B**), and neutrophil (**Figure 2C**) recruitment to the knee joint. As only budlein at 10 mg/kg was able to reduce MSU-induced mechanical hypersensitivity, edema, and leukocyte recruitment, this dose was chosen for the following experiments. Furthermore, using H&E staining for the histopathological analysis of knee joint tissue, we showed that mice treated with budlein A presented reduced MSU-induced synovitis (**Figures 3A–F**) with a lower amount of leukocyte infiltration (**Figure 3G**). LysM-eGFP mice were used as another approach to investigate MSU-induced neutrophil recruitment since eGFP expression is controlled by the promoter of LysM, which is expressed by neutrophils. Treatment with budlein A reduced neutrophil infiltrate (**Figures 4A–G**) as observed by a reduced percentage of fluorescence in confocal microscope (**Figure 4H**). Ultimately, we also found a reduction in MPO activity (**Figure 4I**), an enzyme that is related to neutrophil activity in gout arthritis (Amaral et al., 2012).

### Budlein A Reduces *in vivo* and *in vitro* MSU-Induced IL-1β and TNF-α Production

**FIGURE 5:**
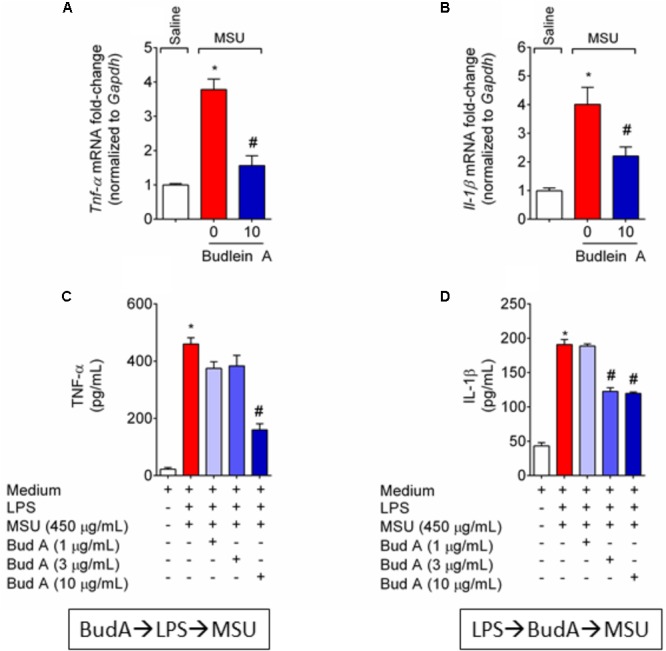
Budlein A reduces *in vivo* and *in vitro* MSU-induced IL-1β and TNF-α production. Fifteen hours after the injection of MSU crystals, knee joint was dissected for the determination of *Tnf-α* **(A)** and *Il-1β* **(B)** mRNA expression by RT-qPCR. Results are expressed as mean ± SEM, *n* = 6 mice per group per experiment, two independent experiments. ^∗^*p* < 0.05 vs. saline group; ^#^*p* < 0.05 vs. 0 mg/kg group, one-way ANOVA followed by Tukey’s *post hoc*. For the *in vitro* assay, BMDMs were pre-treated with budlein A at 1, 3, or 10 μg/mL 30 min before stimulus with LPS for the determination of TNF-α levels **(C)** by ELISA. For the determination of IL-1β levels **(D)** by ELISA, LPS-primed BMDMs were treated with budlein A at 1, 3, or 10 μg/mL 30 min after stimulus LPS. Supernatants were collected 5 h after MSU **(C,D)**. ^∗^*p* < 0.05 vs. saline group; ^#^*p* < 0.05 vs. 0 mg/kg group, one-way ANOVA followed by Tukey’s *post hoc*.

Next, it was evaluated the effect of budlein A in the production of the pro-hyperalgesic cytokines IL-1β and TNF-α in both *in vivo* and *in vitro*. Treatment with budlein A was able to reduce *Il-1β* (**Figure 5A**) and *Tnf-α* (**Figure 5B**) mRNA expression in the knee joint of MSU-stimulated mice.

MSU crystals induce inflammasome assembly and production of mature IL-1β (Martinon et al., 2006). As IL-1β production in its mature form depends on 2 signals (Martinon et al., 2006; Latz et al., 2013) it was next investigated whether the reduction of IL-1β production was related to the inhibition of NF-κB activation (signal 1) or whether budlein A can interfere with inflammasome assembly (signal 2). BMDM pre-treated with budlein A at the concentration of 10 μg/mL reduced MSU-induced TNF-α production (**Figure 5C**), which demonstrates an effect over signal 1 and indicating a possible effect on NF-κB. Treatment with budlein A at the concentrations of 3 and 10 μg/mL reduced MSU-induced IL-1β production in LPS-primed BMDM (**Figure 5D**), indicating that budlein A interferes with signal 2, the inflammasome assembly. We and others have previously shown that the concentration used in this study does not induce cell death (Nicolete et al., 2009; Zarpelon et al., 2017). For instance, budlein A at the concentration of 100 μg/mL reduces cell viability in 18%, but lower concentration such as 10 μg/mL, as the one used in this work, does not affect cell viability (Nicolete et al., 2009; Zarpelon et al., 2017).

### Budlein A Reduces MSU-Induced NLRP3 Inflammasome and NF-κB Activation

**FIGURE 6:**
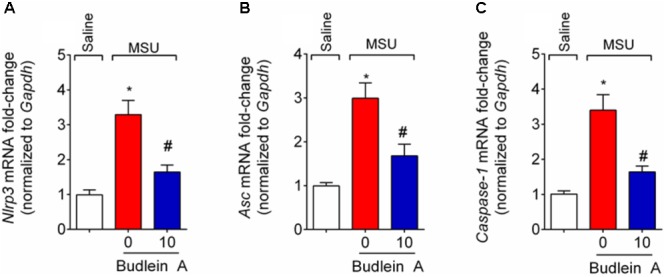
Budlein A inhibits MSU-induced inflammasome assembly. Fifteen hours after the injection of MSU crystals, knee joint was dissected for the determination of *Nlrp3* **(A)**, *Asc* **(B)**, and *Caspase-1* **(C)** mRNA expression by RT-qPCR. ^∗^*p* < 0.05 vs. saline group; ^#^*p* < 0.05 vs. 0 mg/kg group, one-way ANOVA followed by Tukey’s *post hoc*.

**FIGURE 7:**
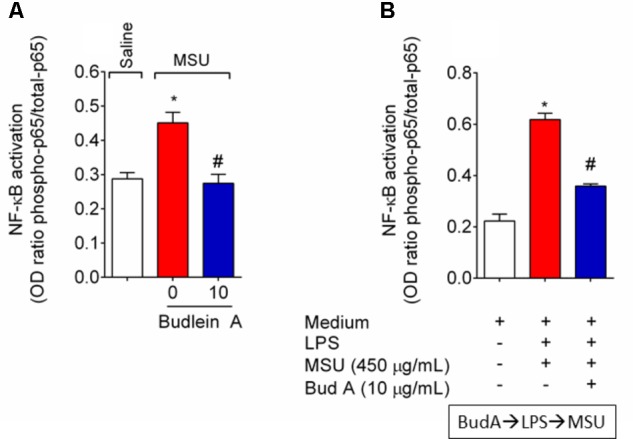
Budlein A reduces MSU-induced NF-κB activation. Fifteen hours after the injection of MSU crystals, knee joint was dissected for the determination of NF-κB activation *in vivo* by ELISA **(A)**. *In vitro*, BMDMs were pre-treated with budlein A at 10 μg/mL 30 min before stimulus with LPS for the determination of NF-κB activation by ELISA **(B)**. BMDMs cell lysates were collected 5 h after MSU **(B)**. ^∗^*p* < 0.05 vs. saline group; ^#^*p* < 0.05 vs. 0 mg/kg group, one-way ANOVA followed by Tukey’s *post hoc*.

Given budlein A reduced IL-1β production (**Figure 5**), we next investigated the effect of budlein A in the mRNA expression of inflammasome components and NF-κB activation after stimulus with MSU. In MSU-stimulated mice, treatment with budlein A at the dose of 10 mg/kg reduced *Nlrp3* (**Figure 6A**), *Asc* (**Figure 6B**), and *Caspase-1* (**Figure 6C**) mRNA expression in the mouse knee joint, and reduced NF-κB activation (**Figure 7A**) as observed by a decrease in the amount of phosphorylated p65 subunit. In corroboration, *in vitro* data also show pre-treated BMDM with budlein A (10 μg/mL) reduced MSU-induced NF-κB activation (**Figure 7B**).

### Budlein A Reduces the Phagocytosis of MSU Crystals by Neutrophils

**FIGURE 8:**
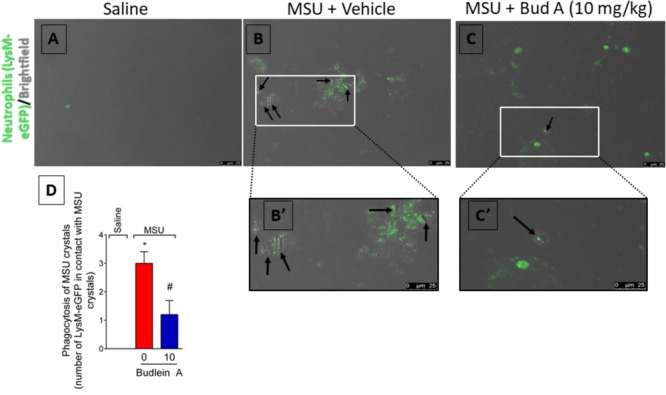
Budlein A reduces phagocytosis of MSU crystals by neutrophils. Fifteen hours after the injection of MSU crystals, knee joint wash of LysM-eGFP mice was collected for quantification of phagocytosis of MSU crystals by neutrophils. Quantification was performed using the brightfield and the green channel using a confocal microscope [Original magnification 63×, panels **(A–C)**]. The representative number of LysM-eGFP positive cells in contact with MSU crystals counted in the 63× objective is presented in panel **(D)**. ^∗^*p* < 0.05 vs. saline group; ^#^*p* < 0.05 vs. 0 mg/kg group, one-way ANOVA followed by Tukey’s *post hoc*.

At the inflammatory foci, recruited neutrophils phagocyte MSU crystals increasing the inflammatory response (Popa-Nita et al., 2007; Mitroulis et al., 2011). To address this issue, it was used knee joint wash from LysM-eGFP mice. *In vivo* imaging of knee joint wash from this mouse strain allows the quantification of the number of neutrophils in contact with MSU crystals, which is not possible by using H&E staining. Here, we show that budlein A decreased the number of LysM-eGFP cells (green channel) in contact with MSU crystals (brightfield) (**Figures 8A–D**). This is an indicative of reduction of MSU crystals phagocytosis upon budlein A treatment. To activate the NLRP3 inflammasome, MSU crystals need first to be phagocyted and then cause the rupture of the phagolysosome releasing cytochalasin that is sensed by NLRP3 (Martinon et al., 2006; Allaeys et al., 2013; Hari et al., 2014; Ruiz-Miyazawa et al., 2017). Therefore, a reduction of phagocytosis would reduce the inflammasome activation.

## Discussion

In this study, we demonstrated the analgesic and anti-inflammatory effects of budlein A in a model of gout arthritis in mice. The injection of MSU crystals in the knee joint of mice resembles an acute gout flare in humans (Amaral et al., 2012; Rees et al., 2014; Dalbeth et al., 2016; Ruiz-Miyazawa et al., 2017). That response is followed by pain, swelling, neutrophil recruitment to the knee joint, and increased levels of pro-inflammatory cytokines, such as IL-1β in a NLRP3 inflammasome-dependent manner (Amaral et al., 2012; Rees et al., 2014; Dalbeth et al., 2016; Ruiz-Miyazawa et al., 2017). We found that treatment with budlein A reduced MSU-induced mechanical hypersensitivity and edema. This inhibition is related to the ability of budlein A in reducing neutrophil recruitment to the knee joint. Further, budlein A reduced *in vivo* and *in vitro* TNF-α and IL-1β production by inhibiting NF-κB activation or interfering with inflammasome assembly.

Pain management of gout acute flare is extremely difficult for clinicians. Currently, it lies in the use of NSAIDs, colchicine, corticoids, or biological agents that often cause severe side effects and lack of safety in patients with comorbidities (Rees et al., 2014; Dalbeth et al., 2016). Therefore, molecules with analgesic properties and fewer side effects are needed. In this sense, naturally occurring molecules have been shown to possess low toxicity and analgesic effect in different models of rheumatic diseases (Khanna et al., 2007; Fattori et al., 2016), including models of gout (Ruiz-Miyazawa et al., 2017). Here, we found that budlein A reduced MSU-induced knee joint mechanical hypersensitivity and edema, which corroborates two reports addressing the analgesic role of budlein A isolated from *V. robusta* (Valerio et al., 2007; Zarpelon et al., 2017). Budlein A analgesic effect is related, at least in part, to the inhibition of NF-κB-dependent mediators. In fact, budlein A ameliorates murine antigen-induced arthritis by inhibiting NF-κB activation and thereby *Il-33, Tnf-α, Il-1β, endothelin-1*, and *Cox-2* mRNA expression in the knee joint (Zarpelon et al., 2017). Moreover, budlein A also reduces carrageenan- and CFA-induced hypersensitivity and pro-inflammatory cytokine productions, such as KC/CXCL1, TNF-α, and IL-1β (Valerio et al., 2007). Importantly, chronic treatment for 7 days with budlein A at 10 mg/kg does not induce liver or stomach damage and *in vitro* data also show that budlein A does not induce cell toxicity at 10 μg/mL (Nicolete et al., 2009; Zarpelon et al., 2017). Altogether, these data suggest that budlein A possesses a safe preclinical profile.

Gout-associated inflammatory response starts with the recognition of MSU crystals by macrophages, which produce IL-1β in an NLRP3 inflammasome-dependent manner (Martinon et al., 2006). In spite of macrophages are the initiators of the MSU crystals-induced inflammatory cascade and neutrophil recruitment plays a major role in bringing inflammation to its full expansion in gout (Martin and Harper, 2010; Amaral et al., 2012; Mitroulis et al., 2013). Seminal studies in crystal-driven gout in canines demonstrated the fundamental role of neutrophils in the acute phase of gout. In these studies, neutrophil depletion significantly suppressed inflammation suggesting that reducing the counts/activity of these cells could be a novel therapeutic approach for gouty arthritis (Phelps and McCarty, 1966; Chang and Garalla, 1968). In fact, neutrophil recruitment is a hallmark of all rheumatic diseases (Fattori et al., 2016) and during gout flares they are the predominant cell population in synovial fluid (Mitroulis et al., 2013; Dalbeth et al., 2016). In contact with MSU crystals, neutrophils degranulate in a Syk-dependent manner (Popa-Nita et al., 2007) and undergo NETosis (Mitroulis et al., 2011), which increase inflammatory response. In the knee joint, activated neutrophil produces and responds to IL-1β, TNF-α, IL-33, and PGE_2_ that ultimately increase pain sensation (Cunha et al., 2008; Verri et al., 2010; Amaral et al., 2012, 2016). These pro-inflammatory mediators are increased in different models of arthritis, such as monoiodoacetate-induced arthritis (Di Cesare Mannelli et al., 2013; Maresca et al., 2017), CFA-induced arthritis (Di Cesare Mannelli et al., 2013; Maresca et al., 2017), and antigen-induced arthritis (Verri et al., 2008; Verri et al., 2010; Zarpelon et al., 2017), indicating that targeting them might contribute to reduce pain. Moreover, strategies aiming at controlling the initial recruitment of neutrophils are interesting approaches for pain relief in gout arthritis. Previous evidence shows that budlein A inhibits neutrophil recruitment (Valerio et al., 2007; Arakawa et al., 2008; Nicolete et al., 2009; Knob et al., 2016; Zarpelon et al., 2017) and activation as observed by a reduction in neutrophil elastase release (Arakawa et al., 2008), an enzyme released in the neutrophil extracellular traps (NETs) (Fattori et al., 2016). In the present work, we demonstrate that budlein A reduced MSU-induced neutrophil recruitment, as observed in the reduced number of LysM-eGFP positive cells in the knee joint, decreased levels of MPO activity, and reduced number neutrophils in the Neubauer chamber. That could be related to the ability of budlein A in reducing the expression of adhesion molecules, such as E-selectin, ICAM-1, and VCAM-1 required for leukocyte transmigration toward tissue, as demonstrated in human umbilical vein endothelial cells (Nicolete et al., 2009). Therefore, the inhibition of neutrophil recruitment represents an important point of the analgesic effects of budlein A.

Macrophages-derived IL-1β also has an important effect on neutrophils. For instance, IL-1β from macrophages stimulates human neutrophils to undergo NETosis after incubation with MSU crystals (Sil et al., 2017). This mechanism possesses a pivotal role in gout acute flares given strategies, such as anakinra (IL-1 receptor antagonist) and colchicines, are used as drugs for pain relief (Rees et al., 2014; Dalbeth et al., 2016). In fact, human neutrophils incubated with Anakinra or anti-IL-1β antibody reduce the release of NETs after stimulus with serum or synovial fluid from patients with gout (Mitroulis et al., 2011). Macrophages produce other mediators, such as macrophage migration inhibitory factor (Galvao et al., 2016) and TNF-α (Amaral et al., 2016), that also drive neutrophil migration toward the tissue. For the production of IL-1β in its mature form, 2 signals are required (Martinon et al., 2006; Latz et al., 2013; Desai et al., 2017). The first signal is related to NF-κB-dependent downstream signaling pathways. While the second signal is related to the inflammasome assembly *per se*, which depends on the phagocytosis of MSU crystals (Martinon et al., 2006; Amaral et al., 2012; Latz et al., 2013). Thus, molecules that target signal 1 or 2 are likely to be highly attractive as an analgesic approach. In corroboration, the flavonoid quercetin that inhibits NLRP3 inflammasome and ASC speck formation (Domiciano et al., 2017) reduces pain and inflammation in experimental gout (Ruiz-Miyazawa et al., 2017). Focusing on budlein A, evidence demonstrates that this SL isolated from *Viguiera arenaria* inhibits NF-κB DNA binding at a concentration of 4.79 μM in TNF-α-stimulated Jurkat T cells (Siedle et al., 2004) and budlein A isolated from *V. robusta* reduces IL-1β- and TNF-α-induced NF-κB activation in RAW 264.7 macrophages expressing NF-κB luciferase gene-reporter (Zarpelon et al., 2017). In addition to targeting NF-κB, the SL parthenolide can direct target inflammasome assembly and thereby inhibiting the maturation of IL-1β (Juliana et al., 2010). Thus, to distinguish the effect of budlein A on both signals, BMDMs were either pre-treated or post-treated regarding LPS stimulus. We found that pre-treated BMDMs (regarding LPS stimulus) reduced MSU-induced TNF-α production, suggesting that budlein A reduced NF-κB activation (signal 1). In addition, budlein A reduced MSU-induced IL-1β production in the supernatant of LPS-primed (post-treatment) BMDMs suggesting that budlein A may also interfere with inflammasome assembly given signal 1 already occurred. Mechanistically, SLs inhibit NF-κB activation by either targeting amino acid residue Cys38 of the NF-κB p65 subunit (Lyss et al., 1998; Widen et al., 2017) or by targeting the upstream enzyme IκB kinase (Siedle et al., 2004; Zhao et al., 2015). Concerning inflammasome assembly, treatment with parthenolide (an α-methylene-γ-lactone) reduces IL-1β maturation by alkylating caspase-1 in the cysteine amino acid residue at position 285 (that is the active site of caspase-1) (Juliana et al., 2010). It is suggested that α-methylene-γ-lactones with an α,β-unsaturated carbonyl structure might share mechanisms of action (Garcia-Pineres et al., 2001; Hohmann et al., 2016). Budlein A is α-methylene-γ-lactones with an α,β-unsaturated carbonyl structure, which is a possible explanation by which budlein A targeted inflammasome assembly. Therefore, direct targeting of inflammasome and reduction of NF-κB activation is likely to explain the *in vitro* and *in vivo* effects of budlein A over TNF-α production and IL-1β production/maturation herein observed (Garcia-Pineres et al., 2001; Hohmann et al., 2016). Therefore, the reduction of MSU-induced IL-1β and TNF-α production and the inhibition of inflammasome components of mRNA expression are crucial to the analgesic effect of budlein A. We also observed a lower number of neutrophils in contact with MSU crystals, indicating reduction in the phagocytosis of MSU crystals. The phagocytosis of crystalline structures, such as the MSU crystals, is also essential to the activation of the inflammasomes (Martinon et al., 2006; Desai et al., 2017). Crystals cause the rupture of the phagolysosome resulting in the release of cytochalasin in the cellular cytoplasm, which will be sensed by the inflammasome (Allaeys et al., 2013; Hari et al., 2014). As this data were observed *in vivo*, the reduced number of cells phagocytosing MSU crystals could be a result of the reduction of neutrophil recruitment to knee joint and also would account to a reduction in inflammasome activation *in vivo* as we showed by RT-qPCR.

Previous work demonstrated that budlein A reduces NF-κB activation and downstream mediators, such as IL-33, TNF-α, IL-1β, and COX-2, in a model of antigen-induced arthritis (Zarpelon et al., 2017). In the present work, we show that the SL budlein A inhibits pain and inflammation in a model of acute gout arthritis in mice. The pathological mechanisms related to gout differ from those in other rheumatic diseases mainly in terms of etiological agent (MSU crystals) and the main immune cells involved in the inflammatory response (macrophages and neutrophils) (Martinon et al., 2006; Martin and Harper, 2010; Amaral et al., 2012; Mitroulis et al., 2013; Dalbeth et al., 2016). The analgesic effect observed herein was related to the inhibition of neutrophil recruitment and IL-1β and TNF-α expression in the knee joint. Moreover, we demonstrated that budlein A decreased MSU-induced maturation of the pro-inflammatory cytokine IL-1β in macrophages. This effect was related to the inhibition of either NF-κB activation (signal 1) or directly targeting inflammasome assembly (signal 2) in these cells. Therefore, molecules that target NF-κB activation and inflammasome assembly, such as budlein A and its analogs, are interesting approaches to treat gout flares.

## Author Contributions

LS-F and TZ treated the animals and injected the stimulus. VF and AZ performed behavioral testing. SB performed confocal microscopy analysis. VF, LS-F, and TZ performed *in vitro* assays. VF, AZ, and WV analyzed and interpreted data set. VF, RC, and WV delineated the study. FC, JA-F, TC, FC, RC, NA, and WV received grants and provided essential reagents. FDC and NA collected plant material, isolated, identified, and purified budlein A. VF and WV wrote, revised, and edited the manuscript. All authors read and approved the final version of the manuscript.

## Conflict of Interest Statement

The authors declare that the research was conducted in the absence of any commercial or financial relationships that could be construed as a potential conflict of interest.
